# The Influence of Acupuncture Parameters on Efficacy and the Possible Use of Acupuncture in Combination with or as a Substitute for Drug Therapy in Patients with Ulcerative Colitis

**DOI:** 10.1155/2022/8362892

**Published:** 2022-03-22

**Authors:** Min'an Chen, Sisi Zhao, Yu Guo, Luxi Cao, Hai Zeng, Zhuowen Lin, Shiqi Wang, Yimin Zhang, Mingmin Zhu

**Affiliations:** School of Traditional Chinese Medicine, Jinan University, Guangzhou 510632, China

## Abstract

**Background:**

Ulcerative colitis (UC) is an inflammatory disease of the colonic mucosa, which is accompanied by chronic, idiopathic characteristics. Acupuncture may be an effective therapy for UC. Here we focused on manual acupuncture and electroacupuncture (MA/EA), two widely used and studied acupuncture interventions, to probe the effects of acupuncture parameters on clinical efficacy in patients with UC and the use of MA/EA alone or with other drugs to support their wider adoption in clinical practice.

**Methods:**

The PubMed, Cochrane Library, Web of Science, Embase, China National Knowledge Infrastructure Database, and Wanfang databases were searched from inception to April 27, 2021. Randomized clinical trials (RCTs) published in Chinese or English were included, and subgroup analyses were performed according to acupuncture parameter, acupuncture type, and control medicine type. The risk of bias was assessed using the Cochrane Risk of Bias tool and modified Jadad scale, and Review Manager 5.4 and Stata 14.0 were used to perform a meta-analysis. Sources of heterogeneity were explored; sensitivity analysis was performed; and the GRADE methodology was used to assess the evidence level.

**Results:**

Sixteen studies (1454 individuals) were included. Retention of the needle [10–30 minutes (RR 1.18, 95% CI [1.11, 1.26], *P* < 0.01; heterogeneity: *χ*^2^ = 6.25, df = 6 (*P*=0.40), I^2^ = 4%)], the frequency of MA [once every other day (RR 1.21, 95% CI [1.08, 1.35], *P* < 0.01; heterogeneity: *χ*^2^ = 0.80, df = 1 (*P*=0.37), I^2^ = 0%)], and the length of treatment [8 weeks (RR 1.35, 95% CI [1.01, 1.81], *P*=0.04)] improved clinical efficacy at the end of treatment compared with medications alone. MA (RR 1.18, 95% CI [1.11, 1.25], *P* < 0.01; heterogeneity: *χ*^2^ = 6.19, df = 7 (*P*=0.52), I^2^ = 0%) increased clinical efficacy compared with medications. Furthermore, MA plus medications (RR 1.26, 95% CI [1.13, 1.40], *P* < 0.01; heterogeneity: *χ*^2^ = 0.95, df = 2 (*P*=0.62), I^2^ = 0%) and EA plus medications (RR 1.36, 95% CI [1.13, 1.63], *P* < 0.01; heterogeneity: *χ*^2^ = 0.13, df = 1 (*P*=0.72), I^2^ = 0%) both dramatically improved clinical efficacy. The clinical efficacy of MA plus mesalazine or MA plus metronidazole and sulfasalazine was greater than with mesalazine or metronidazole and sulfasalazine alone. Similarly, EA plus sulfasalazine was more effective than sulfasalazine alone. MA/EA resulted in fewer adverse reactions than medical therapies. The use of MA plus medications significantly reduced Baron scores. GRADE evaluations indicated that the evidence strength was moderate to low but mostly low.

**Conclusions:**

Our study provides the latest evidence to allow us to speculate about the possible optimal MA parameters to treat patients with UC. The low number of adverse reactions and high efficacy make MA/EA a possible supplement to or replacement for traditional UC drugs. The variable parameter settings preferred by patients and acupuncturists may be an important factor limiting the wider clinical deployment of acupuncture as a potential UC therapy.

## 1. Introduction

Ulcerative colitis (UC) is a chronic, idiopathic inflammatory disease occurring on the colonic mucosa [1], with a global incidence between 0.5 and 24.5 per 100,000 people [2]. The etiopathogenesis of UC remains unclear, but it manifests clinically with abdominal pain, diarrhea, tenesmus, and rectal bleeding [1, 3]. Patients with UC require continuous care and medication [2] due to the persistent and chronic nature of the disease [4]. Furthermore, UC patients are more likely to develop colorectal cancer than the general population [1]. As a result, UC incurs a massive burden on body and mind, quality of life, and healthcare resources.

5-ASA and corticosteroids are common first-line therapies [2] for patients with mild to moderate UC [5]. However, these drugs are associated with side effects, some of which can be severe [6, 7]. Despite their benefit, poor drug compliance can result in patients discontinuing treatment and ultimately poor disease control.

Therefore, there has been increasing interest in complementary and alternative medicines (CAMs) for the treatment of UC, of which traditional Chinese medicine (TCM) is one. [8]. As a natural CAM with an excellent safety profile and few side effects, acupuncture is increasingly recognized as a viable adjunct to other management strategies in many Western countries [9, 10]. The Chinese have used acupuncture to treat UC since ancient times, and it has been revealed to be effective in clinical trials [11–13]. However, the mechanism underpinning the clinical effectiveness of acupuncture in UC patients is still not completely understood, although proposed mechanisms include modulation of gastrointestinal motility, visceral sensitivity, the neuro-endocrine-immune axis, inflammation, and the brain-gut axis [14].

Previous reviews [15] and meta-analyses [16, 17] have focused on the clinical efficacy and adverse reactions of comprehensive acupuncture for UC and confirmed the benign effect of acupuncture for UC, but none of them specifically studied the influence of acupuncture parameters on clinical efficacy, despite them playing an important role in clinical efficacy. In a review, Zhang et al. noted that most current acupuncture studies do not meet dose and quality adequacy criteria for optimal clinical efficacy, including acupuncture manipulation, acupuncture time, frequency, waveform, and other parameters [18]. The acupuncture dosage has always been of importance in TCM, but acupuncture is often practiced based on the beliefs and habits of acupuncturists or even patient preference, significantly restricting the robust exploration and standardization of acupuncture dosage. Nevertheless, scientific advances have allowed in-depth studies of acupuncture dosages and their effect using modern techniques such as the combination of imaging with biochemistry, physiology, and data mining analysis, and animal and clinical studies have also been conducted [19–21]. Fang et al. showed that the differential effects of electroacupuncture (EA) on NTS neuron excitability in normal rats may be caused by different combinations of acupoint and frequency selection [22]. Furthermore, different effects on gastric electrical frequency and amplitude in bradygastria rabbits were related to different manual acupuncture (MA) manipulations of acupoint ST36 [23]. In their in vivo studies, Yang et al. showed that not only needle retention, but also treatment frequency and needle manipulation, were significant determinants of hippocampal learning, memory, and neuron damage in VD rats [24]. In their clinical trial, Xu et al. showed that different acupuncture stimulus techniques had different effects on blood flow perfusion at acupoints in normal adults [25], and in another clinical trial MA stimulation at different acupoints caused different depressor and bradycardic responses [26]. All these data indicate that different acupuncture parameters can produce different clinical effects [27], and acupuncture parameters may be the main factor affecting acupuncture efficacy [28, 29]. Although a growing number of studies have focused on the influence of acupuncture parameters on clinical efficacy [30–32], acupuncture parameters have yet to be thoroughly studied in the clinical management of UC, relevant UC guidelines do not provide detailed acupuncture programs and parameters, and there is still no meta-analysis of the specific clinical impact of acupuncture parameters on UC. Hence, we conducted this systematic evaluation and meta-analysis to probe the optimal MA/EA parameters for the treatment of UC to provide a reference for improvements in the clinical management of UC with MA/EA.

## 2. Materials and Methods

This systematic review and meta-analysis were registered in the International Platform of Registered Systematic Review and Meta-analysis Protocols (INPLASY202190041). The meta-analysis was conducted according to the PRISMA 2020 statement [33].

### 2.1. Eligibility Criteria

To ensure the quality of the meta-analysis, participants, interventions, comparisons, outcomes, and study design (PICOS) approach was adopted.

#### 2.1.1. Inclusion Criteria

  P: The diagnosis of UC was established on the basis of the internationally or nationally recognized diagnostic guideline, which was not less than one (guidelines). Such as the American Gastroenterological Association Clinical Practice Guidelines on the Management of Ulcerative Colitis [34, 35] or the Consensus on TCM Diagnosis and Treatment of Ulcerative Colitis [36]. Participants were 18 years old or older and were not limited by race, gender, geographic location, or disease course.
  I: Manual acupuncture or electroacupuncture (any acupuncture needle specification, acupoint, duration of acupuncture, treatment frequency, period of treatment, and stimulation method) alone or combined with medicines for UC.
  C: Do not treat or wait for treatment, conventional drugs, sham acupuncture, or placebo. When a combination of acupuncture and drugs was used, the drugs in the control group were the same as those in the corresponding experimental group.
  O: The primary outcome was the effective ratio. Secondary outcomes included the adverse effects, Baron scores.
  S: Only randomized controlled trials were eligible.

#### 2.1.2. Exclusion Criteria

The following conditions were not eligible for inclusion: head needle, abdominal needle, ear needle, eye needle, and other non-traditional manual needle therapy; pregnant or lactating patients or those about to become pregnant; patients with mental illness; severe adverse effects of acupuncture (e.g., fear of acupuncture, fainting during acupuncture); animal experiments, case reports, review articles and repeated publications.

### 2.2. Information Sources and Search Strategy

PubMed, Cochrane, Web of Science, Embase, China National Knowledge Infrastructure Database, and Wanfang were searched for all relevant literature from database inception to April 27, 2021. The search strategy was divided into clinical status (UC), intervention (MA/EA), and study type (RCT). We combine Medical Subject Headings (MeSH) and related free text words to search. Differences were resolved through discussion between investigators to reach an agreement. The search details for each database are detailed in Supplementary S1. Moreover, additional publications were identified, which were achieved through manual searching of previously published studies and the reference lists of the included studies.

### 2.3. Data Extraction and Collection

In this process, the duplicates were first removed by two investigators (Min'an Chen and Sisi Zhao) by reading the titles and abstracts. Then, the titles, abstracts, and keywords of the remaining articles were selected and recorded by each investigator individually, according to the inclusion and exclusion criteria. A standardized data extraction form was used to extract general information independently. Missing data or parts related to missing data were removed and not included in the analysis. Furthermore, any differences arising during this process were resolved through negotiation between two investigators (Min'an Chen and Sisi Zhao). If no agreement was reached, a third investigator (Yu Guo) made the final choice to resolve the disagreement.

If different publications included the same participants, the article with the most complete information and the longest follow-up period was selected.

### 2.4. Risk of Bias Assessment

Two authors (Min'an Chen and Sisi Zhao) independently provided an assessment of the risk of bias using the Cochrane Handbook v.5.3.0-recommended Cochrane Risk of Bias assessment (RoB) tool and the modified Jadad quality scale. The RoB assessment tool has six components: random sequence generation, allocation concealment, blinding of participants and personnel, blinding of outcome assessment, incomplete outcome data, selective reporting, and other biases. Risk grade consists of three parts: low bias risk, unclear bias risk, and high bias risk. The modified Jadad quality scale is scored between 1 and 7, with low quality indicated by 1 to 3 and high quality from 4 to 7. Disagreements were resolved by a third investigator (Yu Guo) to reach a consensus.

### 2.5. Statistical Analysis

We analyzed and consolidated the data using the Cochrane Collaboration's Review Manager 5.4 software and Stata 14.0. Two-sided tests were used, and a *P* value <0.05 was considered statistically significant [37, 38]. Relative risk ratios (RR) and corresponding 95% confidence intervals (CIs) were calculated for dichotomous variables. For continuous variables, standard mean differences (SMD) were used to represent the corresponding 95%CIs. Statistical heterogeneity of each trial was evaluated by Cochran's Q statistic and its associated *P* value. In addition, according to the Cochrane Handbook, the I^2^ statistic was selected to test heterogeneity, where a *P* < 0.1 and *I*^2^ ≥ 50% were regarded as high heterogeneity and a random-effects model was used. A *P* < 0.1 and *I*^2^ < 50% were regarded as some heterogeneity and a *P* ≥ 0.1 and *I*^2^ < 50% were considered homogeneous, in which cases fixed-effects models were adopted.

### 2.6. Subgroup Analysis and Sensitivity Analysis

Subgroup analyses of MA/EA parameters, acupuncture type, medicine type in the control group, adverse events, and Baron score were conducted. The robustness of the results was assessed by sensitivity analysis.

### 2.7. Reporting Bias Assessment

The reporting bias was assessed by RevMan version 5.4 and STATA 14.0, which was accomplished by using Funnel plots [39] and Egger's test [40]. If *P* > 0.05 on both sides, there was no reporting bias according to Egger's test.

### 2.8. Confidence Assessment

The evidence level of the outcomes was assessed, which used the Grading of Recommendations Assessment, Development and Evaluation (GRADE) framework [41] by two independent authors (Min'an Chen and Sisi Zhao) (Supplementary S3). The third investigator (Yu Guo) resolved the disagreements to reach a consensus.

## 3. Results

### 3.1. Literature Search

**Figure 1:**
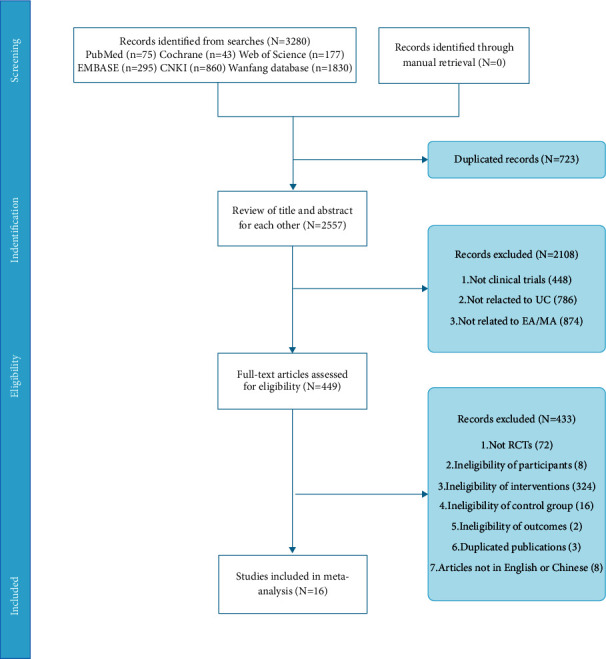
The study selection process.

According to the search strategy, 3280 references were identified and 723 duplicates were excluded. After title and abstract screening, 2108 non-clinical studies and literature unrelated to UC and MA/EA were excluded. Further evaluation of this literature was carried out, and non-RCTs, duplicate publications, non-English papers, and non-Chinese papers were removed, leaving 16 papers for study inclusion after reading the full text.  Figure 1 summarizes the study details, which relate to the selection process.

### 3.2. Study Characteristics

Participants who met the diagnostic criteria for UC were recruited. Sample sizes ranged from 50 [42, 43] to 196 [44].  Table 1 shows the characteristics of the included studies, which included diagnostic criteria, experimental groups, and control groups.

In the experimental group, MA/EA was slightly different with respect to acupoint selection and operation parameters. Thirteen [42–54] trials used a standardized treatment regimen, each with fixed points selected, mainly on the abdomen, back, and lower limbs. Three [49, 55, 56] used semi-standardized treatment schemes, and acupoints were selected according to diagnosis and symptom differentiation using the main acupoints. Among the 14 standardized treatment plans, the main acupoints were Tianshu (ST25), Qihai (RN6), Guanyuan (RN4), Shangjuxu (ST37), and Dachangshu (BL25). Twelve [43–46, 48, 49, 52–57] trials used MA and four [42, 47, 50, 51] used EA. The acupuncture retention time was between 10 min [52, 54] and 60 min [42, 47, 50], and the most common retention time was 30 min [43, 45, 46, 48, 53, 56]. Acupuncture was administered once a day in nine studies [42, 43, 47–50, 53, 55, 56] , five times a week in three studies [42, 47, 50], and every other day in four studies [46, 51, 52, 54]. The total duration of acupuncture treatment ranged from 10 days [49] to 2 months [42, 45, 47, 50], and the median duration of treatment was 30 days [51, 54, 56].

### 3.3. Outcomes Evaluated

Fourteen of 16 trials evaluated clinical efficacy [42–49, 51–56], and 11 studies reported adverse reactions [42, 43, 46–50, 52, 54, 55, 57]. One trial [47] measured ACTH levels, and the other [51] conducted a patient satisfaction survey. One [43] recorded colonoscopic changes, one [46] collected the levels of TNF-*α* and IL-10, and one [45] measured T cell subsets (CD3, CD4, CD8, CD4/CD8). One trial [44] used the Hospital Anxiety and Depression Scale (HADS) scale and measured the disease activity index and serum matrix metalloproteinase (MMP)-9 and trimethylamine N-oxide (TMAO) levels. One trial [57] used Mayo scoring and two trials [44, 46] used Baron scoring to assess disease severity. Two trials [46, 48] measured serum IL-6 and IL-8 levels. Sixteen trials gathered data at the beginning and end of the intervention, and only one [49] collected data only at the end of the intervention. Curiously, none of the trials collected follow-up data after treatment.

### 3.4. Risk of Bias

**Figure 2:**
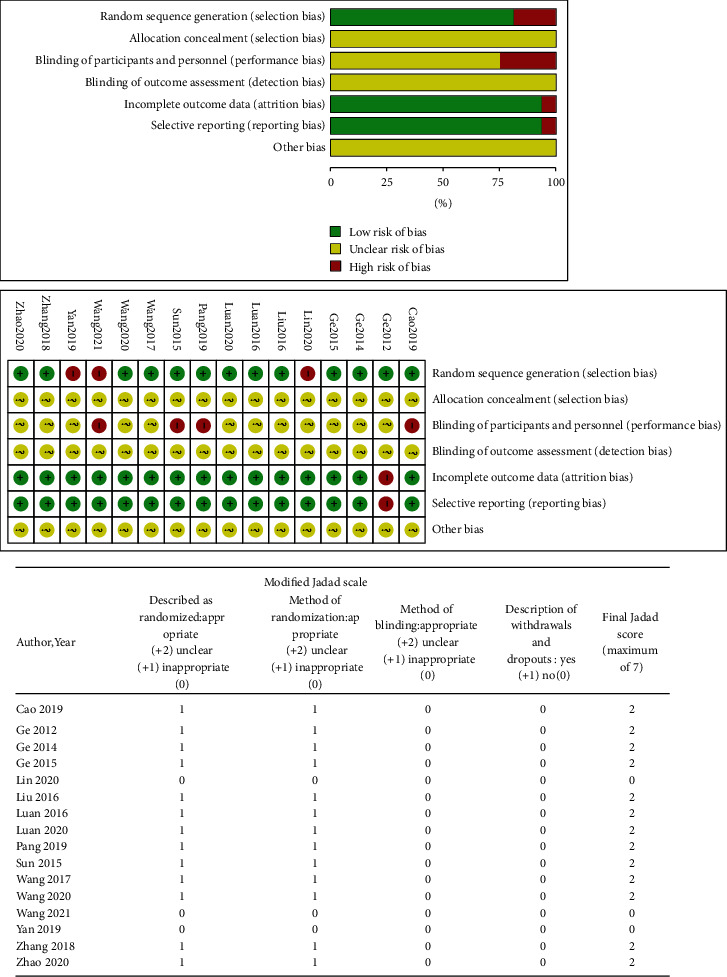
Risk of bias in the included studies.

Graphical summaries of the risk of bias in the included studies are shown in Figure 2.

#### 3.4.1. Cochrane RoB Tool

The main source of bias risk was related to allocation concealment, blinding of participants and personnel, blinding of outcome assessment, and others. A low risk of bias was not present in any of the areas assessed in all trials. Twelve trials [42, 43, 45–49, 52–55, 57] were low risk in three bias risk areas. No item was mentioned for allocation concealment, blinding of outcome assessment, and other bias (Figures 2(a) and 2(b)).

*(1) Random sequence generation*. Thirteen trials had a low risk of randomization bias, and the remaining three trials [44, 51, 56] did not describe the randomization method.

*(2) Allocation concealment*. None of the trials reported assigning hidden methods, so we assessed their risk as “unclear.”

*(3) Blinding of participants and personnel and outcome assessment*. Four trials [44–46, 55] mentioned that patients were informed of treatment, which we assessed as “high risk.” The remaining 12 trials did not mention the blinding of patients and participants and were therefore assessed as “unclear risk.” No trial described whether the outcome assessment was blind and were assessed as “unclear risk.”

*(4) Incomplete outcome data*. One trial [50] had incomplete results, so it was assessed as “high risk.”

*(5) Selective reporting*. Fifteen trials reported all data included in the results and had a low risk of bias. Only one [50] failed to report all pre-stated outcomes and were assessed as “high risk.”

*(6) Other potential sources*. All trials did not describe other potential bias risks and were assessed as “one-sided risk.”

#### 3.4.2. Modified Jadad Scale

Thirteen studies [42, 43, 45–50, 52–55, 57] were of low quality and three were rated 0 [44, 51, 56] (Figure 2(c)).

### 3.5. Primary Outcome

#### 3.5.1. Acupuncture Parameters

**Figure 3:**
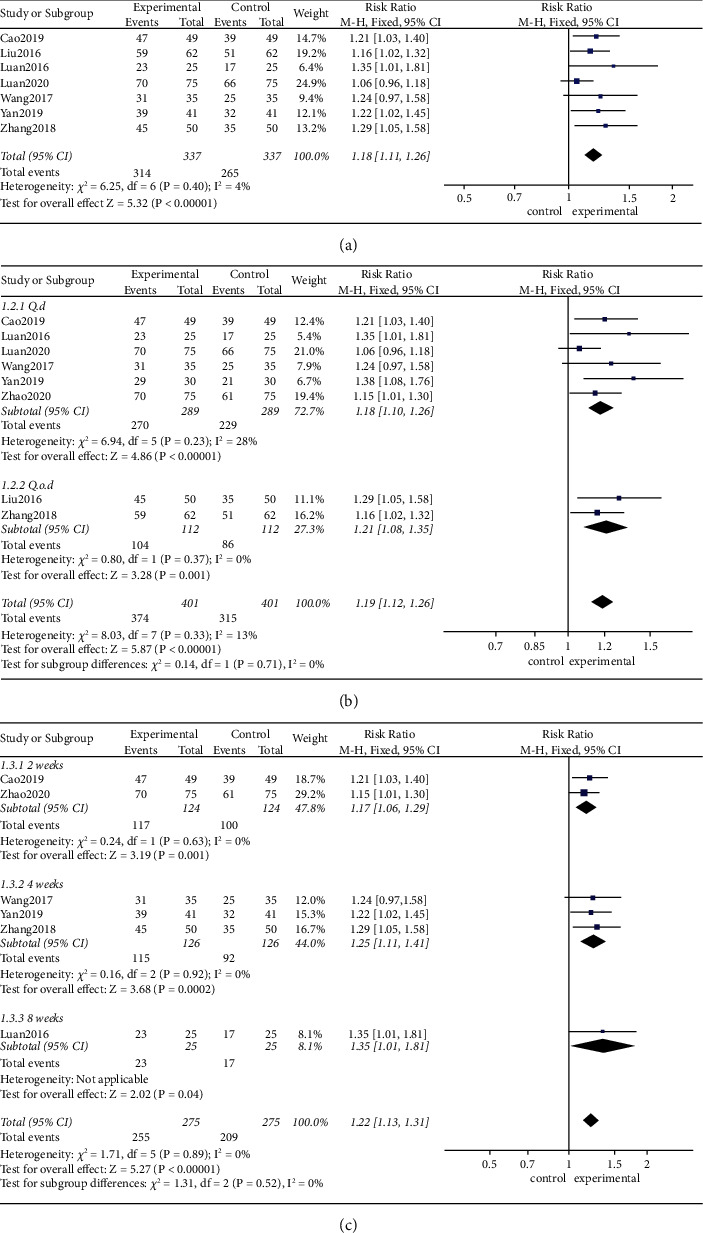
Impact of MA on clinical efficacy. (a) Effects of 10–30 minutes of acupuncture. (b) Effects of acupuncture frequency (once a day and once every other day). (c) Effects of a period of treatment (2 weeks, 4 weeks, and 8 weeks).

The pooled results shown in Figure 3 show the impact of MA parameters on efficacy.

*(1) Duration of acupuncture*. The pooled results shown in Figure 3(a) show that, using a fixed-effects model, retention of the needle for 10–30 minutes (RR 1.18, 95% CI [1.11, 1.26], *P* < 0.01; heterogeneity: *χ*^2^ = 6.25, df = 6 (*P*=0.40), I^2^ = 4%) improved clinical efficacy at the end of treatment compared with medication.

*(2) Acupuncture frequency*. The pooled results shown in Figure 3(b) demonstrate that, using a fixed-effects model, compared with the control group, the frequency of MA [once a day (RR 1.18, 95% CI [1.10, 1.26], *P* < 0.01; heterogeneity: *χ*^2^ = 6.94, df = 5 (*P*=0.23), *I*^2^ = 28%) or once every other day (RR 1.21, 95% CI [1.08, 1.35], *P* < 0.01; heterogeneity: *χ*^2^ = 0.80, df = 1 (*P*=0.37), *I*^2^ = 0%)] both improved clinical efficacy at the end of treatment.

*(3) Period of treatment*. As shown in Figure 3(c), using a fixed-effects model, compared with the control group, the period of treatment [2 weeks (RR 1.17, 95% CI [1.06, 1.29], *P* < 0.01; heterogeneity: *χ*^2^ = 0.24, df = 1 (*P*=0.63), *I*^2^ = 0%), 4 weeks (RR 1.25, 95% CI [1.11, 1.41], *P* < 0.01; heterogeneity: *χ*^2^ = 0.16, df = 2 (*P*=0.92), *I*^2^ = 0%), and 8 weeks (RR 1.35, 95% CI [1.01, 1.81], *P*=0.04)] all improved clinical efficacy at the end of treatment.

#### 3.5.2. Acupuncture Type

**Figure 4:**
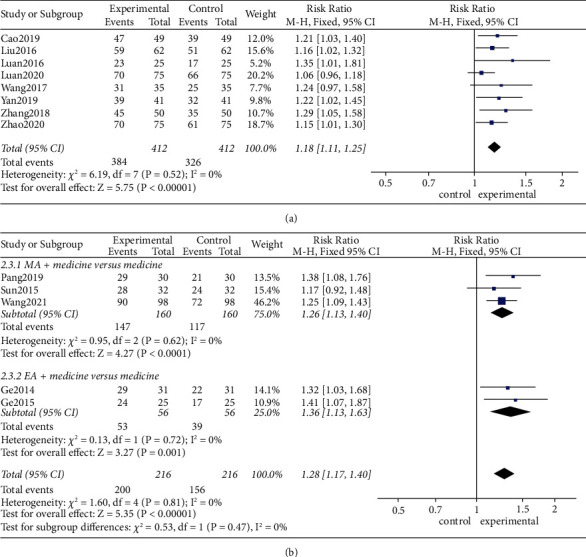
(a) Effects of MA versus medicines on clinical efficacy. (b) Effects of MA plus medicines versus medicines and EA plus medicines versus medicines on clinical efficacy.

As shown in Figure 4, using a fixed-effects model, MA (RR 1.18, 95% CI [1.11, 1.25], *P* < 0.01; heterogeneity: *χ*^2^ = 6.19, df = 7 (*P*=0.52), *I*^2^ = 0%) increased clinical efficacy compared with medicines alone (Figure 4(a)). Furthermore, MA plus medicines (RR 1.26, 95% CI [1.13, 1.40], *P* < 0.01; heterogeneity: *χ*^2^ = 0.95, df = 2 (*P*=0.62), *I*^2^ = 0%) and EA plus medicines (RR 1.36, 95% CI [1.13, 1.63], *P* < 0.01; heterogeneity: *χ*^2^ = 0.13, df = 1 (*P*=0.72), *I*^2^ = 0%) both dramatically improved clinical efficacy (Figure 4(b)).

#### 3.5.3. Type of Medical Therapy

**Figure 5:**
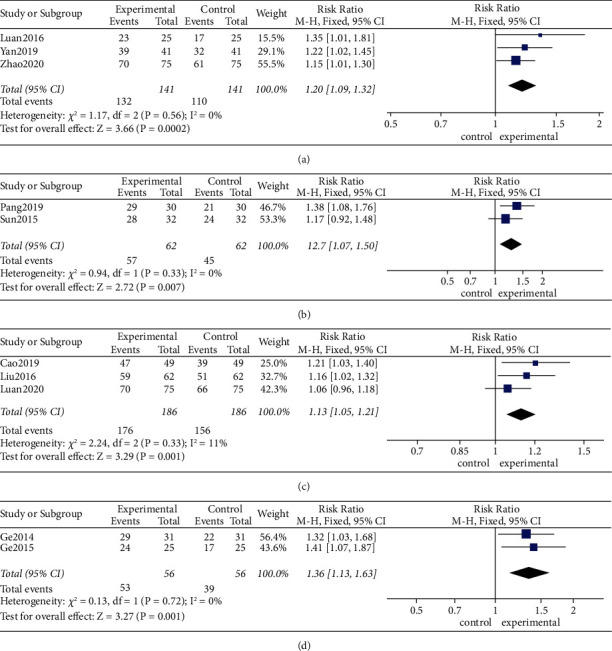
(a) Effects of MA versus mesalazine on clinical efficacy. (b) Effects of MA plus mesalazine versus mesalazine on clinical efficacy. (c) Effects of MA plus (metronidazole + sulfasalazine) versus metronidazole + sulfasalazine on clinical efficacy. (d) Effects of EA plus sulfasalazine versus sulfasalazine on clinical efficacy.

As shown in Figure 5, using a fixed-effects model, the clinical efficacy of MA (RR 1.20, 95% CI [1.09, 1.32], *P* < 0.01; heterogeneity: *χ*^2^ = 1.17, df = 2 (*P*=0.56), *I*^2^ = 0%) alone was greater than oral mesalazine at the end of the intervention (Figure 5(a)). MA plus mesalazine (RR 1.27, 95% CI [1.07, 1.50], *P* < 0.01; heterogeneity: *χ*^2^ = 0.94, df = 1 (*P*=0.33), *I*^2^ = 0%) increased clinical efficacy compared with oral mesalazine (Figure 5(b)). MA plus metronidazole and sulfasalazine (RR 1.13, 95% CI [1.05, 1.21], *P* < 0.01; heterogeneity: *χ*^2^ = 2.24, df = 2 (*P*=0.33), *I*^2^ = 11%) increased clinical efficacy compared with oral metronidazole and sulfasalazine (Figure 5(c)). Similarly, EA plus sulfasalazine (RR 1.36, 95% CI [1.13, 1.63], *P* < 0.01; heterogeneity: *χ*^2^ = 0.13, df = 1 (*P*=0.72), *I*^2^ = 0%) was more effective than oral sulfasalazine (Figure 5(d)).

### 3.6. Secondary Outcomes

#### 3.6.1. Adverse Events

**Figure 6:**
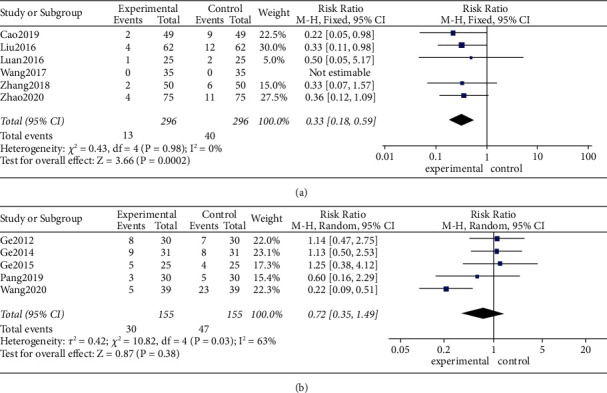
(a) Effects of MA/EA versus medicines on adverse events. (b) Effects of MA/EA plus medicines versus medicines on adverse events.

As shown in Figure 6(a), using a fixed-effects model, use of MA/EA (RR 0.33, 95% CI [0.18, 0.59], *P* < 0.01; heterogeneity: *χ*^2^ = 0.43, df = 4 (*P*=0.98), *I*^2^ = 0%) resulted in fewer adverse reactions than medical therapies. However, in the pooled random-effects model results shown in Figure 6(b), compared with medicines, MA/EA plus medicines (RR 0.72, 95% CI [0.35, 1.49], *P*=0.38; heterogeneity: *χ*^2^ = 10.82, df = 4 (*P*=0.03), *I*^2^ = 63%) had no significant impact on adverse events.

#### 3.6.2. Baron Scores

**Figure 7:**
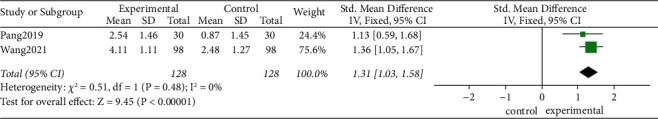
Effects of MA plus medicines versus medicines on baron scores.

As shown in Figure 7, using a fixed-effects model, use of MA plus medicines (RR 1.31, 95% CI [1.03, 1.58], *P* < 0.01; heterogeneity: *χ*^2^ = 0.51, df = 1 (*P*=0.48), *I*^2^ = 0%) significantly reduced Baron scores.

### 3.7. Publication Bias

The pooled results shown in Supplementary S2: Figures S1–S5 show an asymmetrical funnel plot and significant Egger's test (10–30 minutes: *P*=0.008; once a day: *P*=0.013) for acupuncture parameters, suggesting that there may be reporting bias, perhaps through the publication of positive results and small sample sizes.

The pooled results shown in Supplementary S2: Figures S6–S8 show an asymmetrical funnel plot and significant Egger's test (*P*=0.005) for acupuncture type with respect to clinical efficacy.

The pooled results shown in Supplementary S2: Figures S9-S10 show a symmetric funnel plot and non-significant Egger's test (*P*=0.801) for the adverse events of MA/EA versus medicines, suggesting no obvious publication bias. Nevertheless, the small amount of included studies may have reduced the accuracy of the results.

By reason of the limited amount of studies included, publication bias for other outcomes was not assessed.

### 3.8. Sensitivity Analysis

**Figure 8:**
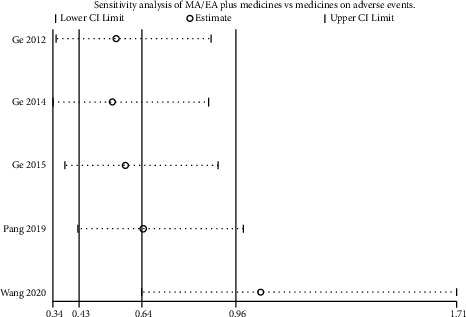
Sensitivity analysis of MA/EA plus medicines vs medicines on adverse events.

**Figure 9:**
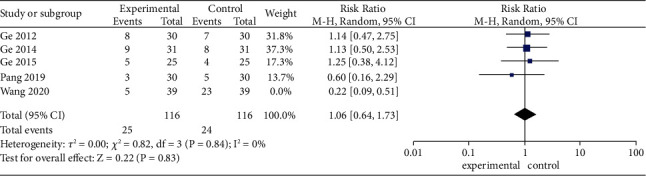
Effects of MA/EA plus medicines versus medicines on adverse events (excluded the study by Wang et al.).

The robustness of the combined results of MA/EA plus medicines vs medicines alone on adverse events was verified by sensitivity analysis (Figure 8), with each included study excluded in sequence. When the study by Wang et al. [47] was excluded (Figure 9), the combined results of MA/EA plus medicines vs medicines on adverse events was not significant (RR 1.06, 95% CI [0.64, 1.73], *P*=0.83; heterogeneity: *χ*^2^ = 0.82, df = 3 (*P*=0.84), *I*^2^ = 0%), suggesting imbalance from this study.

## 4. Discussion

Here we focused on MA/EA, two widely used acupuncture interventions across the world, to explore the influence of their administration parameters on clinical efficacy in patients with UC and the advantage of using them with the usual standard of care drugs to support the promotion and application of MA/EA in clinical practice.

### 4.1. Outcomes

#### 4.1.1. Primary Outcomes

Our study suggests that the impact of MA/EA in patients with UC may be related to the operation parameters used. We therefore explored the impact of the duration of acupuncture retention, frequency of treatment, and duration of treatment in subgroup analyses. With respect to the duration of acupuncture retention, 10–30 minutes significantly enhanced the clinical effect. One treatment every other day seemed to have a slight advantage over daily treatment, and 8 weeks of acupuncture had a slight advantage over shorter treatments in improving the clinical symptoms of UC. Therefore, we hypothesize that 10–30 minutes of acupuncture retention, every other day for 8 weeks, probably represents the optimal protocol informed by existing evidence for the application of MA to patients with UC. In addition, only one study [51] used EA alone as an intervention, with a frequency of 4 times per second, a retention time of 20 minutes, and a treatment course of 30 days once every other day. The results showed clinical efficacy was superior in the acupuncture group than in the medicine group (*P* < 0.01).

Both MA and EA further improved the clinical symptoms and clinical efficacy in UC patients taking pharmaceutical therapies. Furthermore, EA appears to have a therapeutic advantage over MA. In addition, MA/EA combined with medicine appears to be effective in UC as a combined approach. In subgroup analyses, MA plus metronidazole and sulfasalazine was more effective than metronidazole and sulfasalazine; EA plus sulfasalazine was more effective than sulfasalazine; and MA was more effective than mesalazine either alone or in combination.

#### 4.1.2. Secondary Outcomes

In the subgroup analysis, MA/EA more effectively reduced adverse reactions than the control group and there was no statistically significant difference in the combined MA/EA plus medicine subgroup. Considering the small sample size and high risk of bias, the subgroup analysis of MA/EA plus medicine needs interpreting with caution.

In terms of endoscope-related index scores, Baron scores for endoscopic severity changed more after MA was given with pharmaceuticals, suggesting a synergistic effect of MA plus pharmaceutical therapies in treating UC.

### 4.2. Strengths of this Review

First, this study was carried out according to PRISMA 2020 guidelines [33] and, using the PICOS framework, we strictly regulated study inclusion to ensure the quality of assessed RCTs. Second, our study focused on acupuncture parameters. Like the dose of a medicine, acupuncture parameters play an important role in the therapeutic outcome. Our results provide a first step towards the standardization of acupuncture protocols and the motivation to ensure uniformity of acupuncture treatment effects, which would help in the application and promotion of acupuncture therapy. Therefore, we focused on the influence of acupuncture parameters on clinical outcomes in patients with UC. Third, we discussed the additive effects of MA/EA when administered with some medicines and their adverse events to explore the potential of acupuncture as combined therapy with regular, standard of care medications. Fourth, we also included the evaluation of endoscopic symptoms, since endoscopy is central to disease monitoring via changes in intestinal mucosa and plays a very important role in the treatment of UC. Fifth, the heterogeneity of the results was “low”, and we included a comprehensive assessment of reporting bias. Finally, the GRADE framework was used to evaluate the overall quality of evidence [41].

### 4.3. Limitations of this Review

Several limitations were found in this meta-analysis. First, the inclusion criteria were strict and the number and sample size of included RCTs were small, which may have biased the results. Second, none of the included trials were conducted outside China, so there was significant publication bias. Nevertheless, this also highlights that acupuncture treatment for UC has not received due attention in clinical practice in other countries and contexts. Third, the included literature was deficient in blinding. At present, due to the way in which acupuncture is administered, blinding is difficult in practice in most studies. Fourth, the acupuncture parameters included in the literature were not comprehensive, which may be related to the subjective nature of treatment by acupuncturists and/or patients resulting in clinical differences in acupuncture parameters. Therefore, only the parameters available in the relevant literature were tentatively analyzed in our study. Fifth, the included literature generally paid little attention to endoscopic features or effects, which might be overcome with further developments in endoscopy. Finally, no study included extended follow-up, so the long-term effects of MA/EA and its parameters on UC are unclear.

### 4.4. Implications for Practice

We found differences in acupuncture parameters between different studies. Previous studies have shown that a satisfactory therapeutic outcome is inseparable from the acupuncture parameters used, and different acupuncture frequencies, waveforms, intensities [58], and durations play an important role in treatment [2, 59, 60]. Although we provide a set of optimal parameters for acupuncture treatment of UC, this can only be considered a preliminary estimate on the strength of a small amount of low quality and biased studies. We suggest that there is a need for MA/EA studies to examine acupuncture methods and techniques [61] including comprehensive evaluations of manipulation, frequency, current intensity, wave pattern, duration of acupuncture, period of treatment, and needle characteristics. Such studies would help to reduce the variable impact of subjective acupuncture factors on clinical outcomes, thus improving clinical efficacy and promoting the development of highly reproducible, evidence-based acupuncture for UC.

Our results suggest that MA/EA is an effective monotherapy, with fewer adverse reactions than conventional drugs. We found that EA has a slight advantage over MA. In addition, MA/EA may be a good complement to, or even a possible replacement for, mesalazine and SASP, which provides the potential for reducing UC drug therapy for UC patients. There are few reports on the use of MA/EA alone in UC, and areas that would benefit from high-quality clinical studies.

MA appears to improve the features of intestinal mucosa inflammation as seen with endoscopy, but further high-quality evidence would be useful. Therefore, we suggest that colonoscopy with the histopathological evaluation of the intestinal mucosa should be included in any study of acupuncture treatment for UC.

### 4.5. Implications for Research

Given the above clinical implications, there is a crying need to improve the quality of clinical trials studying the acupuncture treatment of UC. We therefore provide the following recommendations. First, any RCT should report according to the CONSORT statement [62] and the reporting standards of acupuncture clinical trials [63]. Second, there needs to be a focus on the operating parameters of acupuncture to establish optimal parameter protocols for clinical deployment. Third, the diagnosis, grading, and inclusion of UC patients should be unified according to common standards, preferably using the endoscopic examination. Fourth, trials must be multi-center, adequately powered, include longer-term follow-up, and actively include non-Chinese institutions to promote the generalizability of results. Finally, it is recommended that clinicians, acupuncturists, endoscopists, examiners, and other stakeholders be consulted during the study design phase to select the best practice plan and reduce the impact of subjective differences on the results.

## 5. Conclusions

In conclusion, our study provides the latest evidence to guide possible optimal parameters for MA: 10–30 min retention, every other day, for 8 weeks. The low number of adverse reactions and high efficacy means that MA/EA can be used as a supplement or even replacement for SASP and mesalazine. Uncertainty over the administration parameters of acupuncture may be an important factor limiting the promotion of acupuncture as a potential UC treatment in clinical practice despite overall evidence of efficacy.

## Figures and Tables

**Table 1:** The main characteristics of the included studies.

Author, Year, Country	Diagnostic criteria	Experimental group										Control group							Outcomes
		Sample size (in each group)	Gender (M/F)	Age (years)	Course of disease	Intervention	Treatment duration					Sample size	Gender (M/F)	Age (years)	Course of disease	Intervention	Treatment duration		
				Mean	Mean		Main acupoints	Duration of acupuncture	Acupuncture frequency	Medication frequency	Total period			Mean	Mean		Medication frequency	Total period	
Cao 2019, China	Diagnosis of chronic UC	49	25/24	28–75	NR	MA	RN12, RN4, DU1, ST25, BL25	20 min	Q.d	NR	2 w	49	26/23	30–75	NR	Metronidazole + SASP	Metronidazole: 0.2 g p.o. T.i.d; SASP: 0.2 g p.o. T.i.d	2 w	ER, AR
				41.4 ± 12.8	NR									40.6 ± 13.2	NR				

Ge 2012, China	Clinical diagnosis and treatment guide	30	31/29 (tot)	19–58 (tot)	2 m–7 y (tot)	EA + SASP	ST25, RN6, RN4, ST37, SP6, LR3; BL18, BL20, BL25, BL23, BL32 (alternate)	60 min	Q.d	The first month: 0.5 g p.o. Q.i.d; the next month: 1.5 g/d	2 m	30	31/29 (tot)	19–58 (tot)	2 m–7 y (tot)	SASP	The first month: 0.5 g p.o. Q.i.d; the next month: 1.5 g/d	2 m	AR
				31.7 ± 4.5 (tot)	(3.8 ± 2.1) *y* (tot)									31.7 ± 4.5 (tot)	(3.8 ± 2.1) *y* (tot)				

Ge 2014, China	Clinical diagnosis and treatment guide	31	16/15	26–71	4 m–7 y	EA + SASP	BL18, BL20, BL25, BL23, BL32, ST25, RN6, RN4, ST37, SP6, LR3 (alternate)	60 min	Q.d	The first month: 1 g p.o. Q.i.d; the next month: 2 g/d	2 m	31	17/14	25–73	5 m–8 y	SASP	The first month: 1 g p.o. Q.i.d; the next month: 2 g/d	2m	ER, AR, ACTH
				35.6 ± 7.5	(3.7 ± 2.8) y									38.4 ± 7.8	(4.0 ± 2.5) y				

Ge 2015, China	Clinical diagnosis and treatment guide	25	27/23 (tot)	27–71 (tot)	5 m–8 y (tot)	EA + SASP	BL18, BL23, BL20, BL25, BL32, ST25, ST37, RN4, RN6, LR3, SP6 (alternate)	60 min	Q.d	The first month: 1 g p.o. Q.i.d; the next month: 2 g/d	2 m	25	27/23 (tot)	27–71 (tot)	5 m–8 y (tot)	SASP	The first month: 1 g p.o. Q.i.d; the next month: 2 g/d	2m	ER, AR
				38.5 ± 6.5 (tot)	(4.1 ± 2.7) *y* (tot)									38.5 ± 6.5 (tot)	(4.1 ± 2.7) *y* (tot)				

Lin 2020, China	Diagnosis of chronic UC	30	8/22	21–77	3 m–13 y	EA	RN12, ST36, ST37, LI11, BL23, BL25	40 min	Q.o.d	NR	30 d	30	11/19	20–73	3 m–15 y	Diphenoxylate Co. + norfloxacin + berberine Co	Diphenoxylate Co.: 2^#^p.o. T.i.d, norfloxacin: 0.2 g 2^#^p.o. T.i.d,; berberine: 3^#^p.o. T.i.d	30 d	ER, SE
				NR	NR									NR	NR				

Liu 2016, China	Diagnosis of chronic UC	62	29/33	23–76	(9–19) m	MA	RN4, RN6, ST25, BL25, DU1	10–30 min	Q.o.d	NR	NR	62	30/32	24–74	(9–20) m	Metronidazole + SASP	Metronidazole: 2^#^-3^#^，T.i.d p.o. SASP: 2-3 g/d T.i.d p.o.	NR	ER, AR
				50.67 ± 6.82	(13.63 ± 5.16) m									51.14 ± 5.46	(14.10 ± 5.22) m				

Luan 2016, China	Diagnosis of chronic UC	25	13/12	26–42	NR	MA	SP4, KI3, ST36, RN4, ST25，BL16, BL20, BL21, BL22, BL25; DU2, DU6	30 min	Q.d	NR	8 w	25	14/11	23–43	NR	Mesalazine	1 g p.o. Q.i.d	8 w	ER, AR
				34 ± 5.75	NR									31.28 ± 6.13	NR				

Luan 2020, China	Diagnosis of chronic UC	75	40/35	24–76	(9–17) m	MA	ST25, RN4, RN6, BL25	30 min	Q.d	NR	NR	75	45/30	25–75	(9–18) m	Metronidazole + SASP	Metronidazole: 0.2 g p.o. T.i.d,; SASP: 2.0 g p.o. T.i.d	NR	ER
				NR	NR									NR	NR				

Pang 2019, China	Consensus opinions on the diagnosis and treatment of inflammatory bowel disease	30	16/14	20–67	(4–68) m	MA + mesalazine	BL31, BL32, BL33, BL34	30 min	Q.o.d	1.0 g p.o. Q.i.d	1 m	30	19/11	20–64	(4–66) m	Mesalazine	1.0 g p.o. Q.i.d	1 m	ER, baron score; serum TNF-*α*, IL-6, IL-8, IL-10
				41.63 ± 12.86	(36.90 ± 20.94) m									43.33 ± 15.51	(38.03 ± 18.42) m				
Sun 2015, China	Consensus opinions on the diagnosis and treatment of inflammatory bowel disease	32	16/16	28–60	≤8 y	MA + mesalazine	RN3, RN4, RN6; ST25, SP15; BL25; ST36, ST37, SP6; LR3	30 min	NR	1 g p.o. Q.i.d	2 m	32	18/14	24–57	≤7 y	Mesalazine	1 g p.o. Q.i.d	2 m	ER, T cell subsets
Wang 2017, China	World gastroenterology organization practice guidelines for the diagnosis and management of IBD in 2010	35	NR	NR	NR	MA	RN12, ST25, RN6, ST36, SP6	30 min	Q.d	NR	4 w	35	NR	NR	NR	SASP	500 mg p.o. Q.i.d	4 w	ER, serum IL-6, IL-8, AR

Wang 2020, China	Diagnosis of chronic UC	39	22/17	44–54	NR	MA + aminosalicylic acid	Sibian, ST25, RN4, RN6	15–20 min	NR	1-2^#^p.o. T.i.d	NR	39	25/14	45–61	NR	Aminosalicylic acid	1-2^#^p.o. T.i.d	NR	Mayo score, AR
				49.27 ± 5.17	NR									53.13 ± 8.23	NR				

Wang 2021, China	Consensus opinions on the diagnosis and treatment of inflammatory bowel disease	98	46/52	−	NR	MA + mesalazine + flupentixton melitoxin	Guiyan (LU11, SP1）	20 min	NR	Mesalazine, 500 g p.o. T.i.d; flupentixton melitoxin (0.5 mg/10 mg), 1^#^p.o. Q.d	1 m	98	44/54	NR	NR	Mesalazine + flupentixton melitoxin	Mesalazine: 500 g p.o. T.i.d; flupentixton melitoxin (0. 5 mg/10 mg): 1^#^p.o. q.d.	1 m	ER, baron score, HADS scale, serum MMP –9, TMAO
				37.32 ± 8.16	(13.14 ± 5.46) m									38.16 ± 9.52	(13.62 ± 6.58) m				

Yan 2019, China	Diagnosis of chronic UC	41	25/16	24–61	NR	MA	ST25, LR13, LI4, BL20, ST37	30 min	Q.d	NR	30 d	41	27/14	25–63	NR	Mesalazine	1 g p.o. Q.i.d	30 d	ER
				42.5 ± 4.4	NR									44.3 ± 4.6	NR				

Zhang 2018, China	Diagnosis of chronic UC	50	26/24	35–69	10 d–3 y	MA	RN4, RN6, ST25, DU1, BL25	10–30 min	Q.o.d	NR	30 d	50	30/20	34–70	9 d–2 y	Metronidazole	0.2 g p.o. T.i.d	30 d	ER, AR
				45.6 ± 0.01	(2.1 ± 0.01) y									46.5 ± 0.5	(1.2 ± 0.01) y				
Zhao 2020, China	Diagnosis of chronic UC	75	35/40	NR	NR	MA	ST36, ST37, ST25, DU1, ST36, SP6, ST25	NR	Q.d	NR	10 d	75	42/33	NR	NR	Mesalazine	p.o. T.i.d	10 d	ER, AR, SF-36

## Abbreviations

M/F: Male/female
y: Year
m: Month
w: Week
d: Day
UC: Ulcerative colitis
tot: Total
MA: Manual acupuncture
EA: Electroacupuncture
SASP: Sulfasalazine
Q.D: Quaque die
T.I.D: Third in die
Q.I.D: Quarter in die
Q.O.D: Quaque omni die
CW: Continuous wave
P.O: Per os
NR: Not reported
ER: Effective rate
AR: Adverse reaction.

## Supplementary Materials

Supplementary Materials Supplementary S1: search strategy for different databases. Supplementary S2: funnel plot and Egger's test graphs of outcomes. Supplementary S3: GRADE Quality evaluation form. Supplementary S4: PRISMA_2020_checklist.
