# Supplementary material for: The Influence of Acupuncture Parameters on Efficacy and the Possible Use of Acupuncture in Combination with or as a Substitute for Drug Therapy in Patients with Ulcerative Colitis
Source: Evid Based Complement Alternat Med. 2022 Mar 22;2022:8362892. doi: 10.1155/2022/8362892 (PMC8964160; doi:10.1155/2022/8362892)
Supplement: Supplementary Materials — Supplementary S1: search strategy for different databases. Supplementary S2: funnel plot and Egger's test graphs of outcomes. Supplementary S3: GRADE Quality evaluation form. Supplementary S4: PRISMA_2020_checklist. [file 8362892.f1.zip › 8362892.f1/SUPPLE_1.doc]

**Supplementary S1**

**Search strategies used for different databases**

**PubMed**

1. Colitis, Ulcerative[MeSH Terms]
2. Colitis[MeSH Terms]
3. Pouchitis[MeSH Terms]
4. Enteritis[MeSH Terms]
5. Proctitis[MeSH Terms]
6. Proctocolitis[MeSH Terms]
7. #1 OR #2 OR #3 OR #4 OR #5 OR #6
8. Coliti*[Title/Abstract]
9. Pouch*[Title/Abstract]
10. Enteriti*[Title/Abstract]
11. Proct*[Title/Abstract]
12. Recto*[Title/Abstract]
13. #8 OR #9 OR #10 OR #11 OR #12
14. #7 OR #13
15. Acupuncture[MeSH Terms]
16. Acupuncture Therapy[MeSH Terms]
17. Acupuncture Points[MeSH Terms]
18. Acupuncture Analgesia[MeSH Terms]
19. Electroacupuncture[MeSH Terms]
20. Meridians[MeSH Terms]
21. #15 OR #16 OR #17 OR #18 OR #19 OR #20
22. Pharmacopunct*[Title/Abstract]
23. Acupunct*[Title/Abstract]
24. Acupoint*[Title/Abstract]
25. Acu-point*[Title/Abstract]
26. Electroacupunct*[Title/Abstract]
27. Meridian*[Title/Abstract]
28. Ching Lo[Title/Abstract]
29. luo, jing[Title/Abstract]
30. Jing Luo[Title/Abstract]
31. Jingluo[Title/Abstract]
32. Jing mai[Title/Abstract]
33. Jingmai[Title/Abstract]
34. #22 OR #23 OR #24 OR #25 OR #26 OR #27 OR #28 OR #29 OR #30 OR #31 OR #32 OR #33
35. #21 OR #34
36. Randomized Controlled Trial[Publication Type]
37. Controlled Clinical Trial[Publication Type]
38. Clinical Trial[Publication Type]
39. Clinical Study [Publication Type]
40. [Multicenter Study[Publication Type]](https://www.ncbi.nlm.nih.gov/mesh/68016448)
41. Comparative Study[Publication Type]
42. #36 OR #37 OR #38 OR #39 OR #40 OR #41
43. Randomized Controlled Trials as Topic[MeSH Terms]
44. Controlled Clinical Trials as Topic[MeSH Terms]
45. Clinical Trials as Topic[MeSH Terms]
46. Clinical Studies as Topic[MeSH Terms]
47. Random Allocation[MeSH Terms]
48. Multicenter Studies as Topic[MeSH Terms]
49. Follow-Up Studies[MeSH Terms]
50. #43 OR #44 OR #45 OR #46 OR #47 OR #48 OR #49
51. random* [Title/Abstract]
52. RCT[Title/Abstract]
53. controll*[Title/Abstract]
54. CCT[Title/Abstract]
55. clinical[Title/Abstract]
56. trial*[Title/Abstract]
57. multicent*[Title/Abstract]
58. allocat*[Title/Abstract]
59. followup*[Title/Abstract]
60. follow up*[Title/Abstract]
61. #51 OR #52 OR #53 OR #54 OR #55 OR #56 OR #57 OR #58 OR #59 OR #60
62. #42 OR #50 OR #61
63. animals[MeSH Terms]
64. humans[MeSH Terms]
65. #63 NOT #64
66. #14 AND #35 AND #62
67. #66 NOT #65

**Embase**

#1 'ulcerative colitis'/exp
#2 'colitis'/exp
#3 'enteritis'/exp
#4 'enterocolitis'/exp
#5 'proctitis'/exp
#6 'proctocolitis'/exp
#7 #1 OR #2 OR #3 OR #4 OR #5 OR #6
#8 coliti*:ti,ab,kw
#9 enteriti*:ti,ab,kw
#10 enterocoliti*:ti,ab,kw
#11 proct*:ti,ab,kw
#12 recto*:ti,ab,kw
#13 #8 OR #9 OR #10 OR #11 OR #12
#14 #7 OR #13
#15 'acupuncture'/exp
#16 'acupuncture point'/exp
#17 'acupuncture Analgesia'/exp
#18 'electroacupuncture'/exp
#19 'body meridian'/exp
#20 #15 OR #16 OR #17 OR #18 OR #19
#21 pharmacopunct*:ti,ab,kw
#22 acupoint*:ti,ab,kw
#23 acupunct*:ti,ab,kw
#24 electroacupunct*:ti,ab,kw
#25 meridian*:ti,ab,kw
#26 'Ching Lo':ti,ab,kw
#27 'luo, jing':ti,ab,kw
#28 (Jing NEAR/1 Luo):ti,ab,kw
#29 'Jingluo':ti,ab,kw
#30 'Jing mai':ti,ab,kw
#31 'Jingmai':ti,ab,kw
#32 #21 OR #22 OR #23 OR #24 OR #25 OR #26 OR #27 OR #28 OR #29 OR #30 OR #31
#33 #20 OR #32
#34 'randomized controlled trial'/exp
#35 'randomized controlled trial topic'/exp
#36 'randomization'/exp
#37 'controlled clinical trial'/exp
#38 'controlled clinical trial topic'/exp
#39 'clinical trial'/exp
#40 'clinical trial topic'/exp
#41 'clinical study'/exp
#42 'multicenter Study'/exp
#43 'multicenter study topic'/exp
#44 'comparative study'/exp
#45 'follow up'/exp
#46 #34 OR #35 OR #36 OR #37 OR #38 OR #39 OR #40 OR #41 OR #42 OR #43 OR #44 OR #45
#47 random*:ti,ab,kw
#48 controll*:ti,ab,kw
#49 'clinical':ti,ab,kw
#50 'rct':ti,ab,kw
#51 'cct':ti,ab,kw
#52 allocat*:ti,ab,kw
#53 trial*:ti,ab,kw
#54 multicent*:ti,ab,kw
#55 comparative stud*:ti,ab,kw
#56 followup*:ti,ab,kw
#57 follow up*:ti,ab,kw
#58 #47 OR #48 OR #49 OR #50 OR #51 OR #52 OR #53 OR #54 OR #55 OR #56 OR #57 OR #58
#59 #46 OR #58
#60 #14 AND #33 AND #59
#61 'animal'/exp
#62 'human'/exp
#63 #61 NOT #62
#64 #60 NOT #63

**Cochrane Library**

#1 MeSH descriptor: [Colitis, Ulcerative] explode all trees
#2 MeSH descriptor: [Colitis] explode all trees
#3 MeSH descriptor: [Enteritis] explode all trees
#4 MeSH descriptor: [Enterocolitis] explode all trees
#5 MeSH descriptor: [Proctitis] explode all trees
#6 MeSH descriptor: [Proctocolitis] explode all trees
#7 #1 OR #2 OR #3 OR #4 OR #5 OR #6
#8 (coliti*):ti,ab,kw
#9 (enteriti*):ti,ab,kw
#10 (enterocoliti*):ti,ab,kw
#11 (proct*):ti,ab,kw
#12 (recto*):ti,ab,kw
#13 #8 OR #9 OR #10 OR #11 OR #12
#14 #7 OR #13
#15 MeSH descriptor: [Acupuncture] explode all trees
#16 MeSH descriptor: [Acupuncture Therapy] explode all trees
#17 MeSH descriptor: [Acupuncture Points] explode all trees
#18 MeSH descriptor: [Acupuncture Analgesia] explode all trees
#19 MeSH descriptor: [Electroacupuncture] explode all trees
#20 MeSH descriptor: [Meridians] explode all trees
#21 #15 OR #16 OR #17 OR #18 OR #19 OR #20
#22 (pharmacopunct*):ti,ab,kw
#23 (acupunct*):ti,ab,kw
#24 (acupoint*):ti,ab,kw
#25 (electroacupunct*):ti,ab,kw
#26 (meridian*):ti,ab,kw
#27 (Ching Lo):ti,ab,kw
#28 (luo, jing):ti,ab,kw
#29 (Jing NEAR/1 Luo):ti,ab,kw
#30 (Jingluo):ti,ab,kw
#31 (Jing mai):ti,ab,kw
#32 (Jingmai) :ti,ab,kw
#33 #22 OR #23 OR #24 OR #25 OR #26 OR #27 OR #28 OR #29 OR #30 OR #31 OR #32
#34 #21 OR #33
#35 (Randomized Controlled Trial):pt
#36 (Controlled Clinical Trial):pt
#37 (Clinical Trial):pt
#38 (Clinical Study):pt
#39 (Multicenter Study):pt
#40 (Comparative Study):pt
#41 #35 OR #36 OR #37 OR #38 OR #39 OR #40
#42 MeSH descriptor: [Randomized Controlled Trial] explode all trees
#43 MeSH descriptor: [Controlled Clinical Trial] explode all trees
#44 MeSH descriptor: [Clinical Trial] explode all trees
#45 MeSH descriptor: [Randomized Controlled Trials as Topic] explode all trees
#46 MeSH descriptor: [Controlled Clinical Trials as Topic] explode all trees
#47 MeSH descriptor: [Clinical Trials as Topic] explode all trees
#48 MeSH descriptor: [Clinical Studies as Topic] explode all trees
#49 MeSH descriptor: [Multicenter Studies as Topic] explode all trees
#50 MeSH descriptor: [Clinical Study] explode all trees
#51 MeSH descriptor: [Multicenter Study] explode all trees
#52 MeSH descriptor: [Comparative Study] explode all trees
#53 MeSH descriptor: [Follow-Up Studies] explode all trees
#54 MeSH descriptor: [Random Allocation] explode all trees
#55 #42 OR #43 OR #44 OR #45 OR #46 OR #47 OR #48 OR #49 OR #50 OR #51 OR #52 OR #53 OR #54
#56 (random*):ti,ab,kw
#57 (RCT):ti,ab,kw
#58 (clinical):ti,ab,kw
#59 (controll*):ti,ab,kw
#60 (CCT):ti,ab,kw
#61 (trial*):ti,ab,kw
#62 (multicent*):ti,ab,kw
#63 (Comparative Stud*):ti,ab,kw
#64 (allocat*):ti,ab,kw
#65 (followup*):ti,ab,kw
#66 (follow up*):ti,ab,kw
#67 #56 OR #57 OR #58 OR #59 OR #60 OR #61 OR #62 OR #63 OR #64 OR #65 OR #66
#68 #41 OR #55 OR #67
#69 #14 AND #34 AND #68

**Web of Science**

#1 TS=( “coliti*” OR “pouch*” OR “enteriti*” OR “proct*” OR “recto*”)
#2 TS=(“acupunct*” OR “pharmacopunct*” OR “acupoint*” OR “acu-point*” OR “electroacupunct*” OR “meridian*” OR “Ching Lo” OR “luo, jing” OR “Jing NEAR/1 Luo” OR “Jingluo” OR “Jingmai” OR “Jing mai”)
#3 TS=(“random*” OR “RCT” OR “clinical” OR “CCT” OR “controll*” OR “trial*” OR “	multicent*” OR “comparative stud*” OR “allocat*” OR “followup*” OR “follow-up*”)
#4 #1 AND #2 AND #3

**CNKI**

(((SU% = 'ulcerative colitis' + 'colitis' + 'UC') OR (TKA = 'ulcerative colitis' + 'colitis' + 'UC'))

AND ((SU% = 'needle' + 'acupuncture' + 'electroacupuncture' + 'manual acupuncture' + 'manually operated needles' + 'body acupuncture' + 'manipulation of the needle' + 'acupuncture therapy' + 'retaining needle') OR (TKA = 'needle' + 'acupuncture' + ' electroacupuncture' + 'manual acupuncture' + 'manually operated needles' +' body acupuncture ' + 'manipulation of the needle' + 'acupuncture therapy' + 'retaining needle' + 'MA' + 'EA')) AND ((SU% = 'RCT') OR (SU% = 'random' + 'control' + 'allocation' + 'clinic' + 'trial' + 'multicenter' + 'follow-up' + 'CCT') OR (TKA = 'random' + 'control' + 'allocation' + 'clinic' + 'trial' + 'multicenter' + 'follow-up' + 'RCT' + 'CCT'))) NOT ((SU%= 'animal experiment') OR (TKA = 'rat') OR (TKA = 'rabbit'))

**Wangfang**

(SU%: ('ulcerative colitis' or 'colitis' or 'UC') and SU%: ('needle' or 'acupuncture' or 'electroacupuncture' or 'manual acupuncture' or 'manually operated needles' or 'body acupuncture' or 'manipulation of the needle' or 'acupuncture therapy' or 'retaining needle' or 'MA' or 'EA') and SU%: ('random' or 'control' or 'allocation' or 'clinic' or 'trial' or 'multicenter' or 'follow-up' or 'RCT' or 'CCT')) not (SU%: ('animal experiment' or 'rat' or 'rabbit'))
