# Supplementary material for: The Influence of Acupuncture Parameters on Efficacy and the Possible Use of Acupuncture in Combination with or as a Substitute for Drug Therapy in Patients with Ulcerative Colitis
Source: Evid Based Complement Alternat Med. 2022 Mar 22;2022:8362892. doi: 10.1155/2022/8362892 (PMC8964160; doi:10.1155/2022/8362892)
Supplement: Supplementary Materials — Supplementary S1: search strategy for different databases. Supplementary S2: funnel plot and Egger's test graphs of outcomes. Supplementary S3: GRADE Quality evaluation form. Supplementary S4: PRISMA_2020_checklist. [file 8362892.f1.zip › 8362892.f1/SUPPLE_2 figures.DOC]

**Supplementary S2:**

Funnel plot and Egger’s test graphs of outcomes.

**Figure 1.** Funnel plot for the clinical efficacy of MA versus medicine (duration of acupuncture for 10-30 minutes).

**Figure 2.** Egger’s test for publication bias of duration of acupuncture for 10-30 minutes was significant (p = 0.008).

**Figure 3.** Funnel plot for the clinical efficacy of MA versus medicine (acupuncture frequency).

**Figure 4.** Egger’s test for publication bias of once a day was significant (p = 0.013).

**Figure 5.** Funnel plot for the clinical efficacy of MA versus medicine (period of treatment).

**Figure 6.** Funnel plot for the clinical efficacy of MA versus medicine.

**Figure 7.** Egger’s test for publication bias of MA versus medicine (clinical efficacy) was significant (p = 0.005).

**Figure 8.** Funnel plot for the clinical efficacy of MA/EA plus medicine versus medicine.

**Figure 9.** Funnel plot for the adverse events of MA/EA versus medicine.

**Figure 10.** Egger’s test for publication bias of MA/EA versus medicine (adverse events) was not significant (p = 0.801).
