# Supplementary material for: The Influence of Acupuncture Parameters on Efficacy and the Possible Use of Acupuncture in Combination with or as a Substitute for Drug Therapy in Patients with Ulcerative Colitis
Source: Evid Based Complement Alternat Med. 2022 Mar 22;2022:8362892. doi: 10.1155/2022/8362892 (PMC8964160; doi:10.1155/2022/8362892)
Supplement: Supplementary Materials — Supplementary S1: search strategy for different databases. Supplementary S2: funnel plot and Egger's test graphs of outcomes. Supplementary S3: GRADE Quality evaluation form. Supplementary S4: PRISMA_2020_checklist. [file 8362892.f1.zip › 8362892.f1/SUPPLE_3 (2).DOC]

**Supplementary** **S3: GRADE Quality evaluation form.**

**Table. 1**

**Summary of findings: The influence of acupuncture parameters on efficacy in patients with ulcerative colitis.**

| **The influence of acupuncture parameters on efficacy in patients with ulcerative colitis.** | | | | | | |
| --- | --- | --- | --- | --- | --- | --- |
| **Patient or population:** patients with UC **Settings:** outpatient/ inpatient **Intervention:** MA **Comparison:** medicine | | | | | | |
| **Outcomes** | **Illustrative comparative risks* (95% CI)** | | **Relative effect (95% CI)** | **No of Participants (studies)** | **Quality of the evidence (GRADE)** | **Comments** |
|  | Assumed risk | Corresponding risk |  |  |  |  |
|  | **Medicine** | **MA** |  |  |  |  |
| **Duration of MA within 30 minutes** | **Study population** | | **RR 1.18**  (1.11 to 1.26) | 674 (7 studies) | ⊕⊕⊝⊝ **low**^1,2^ |  |
|  | **786 per 1000** | **928 per 1000** (873 to 991) |  |  |  |  |
|  | **Moderate** | |  |  |  |  |
|  | **781 per 1000** | **922 per 1000** (867 to 984) |  |  |  |  |
| **MA frequency** | **Study population** | | **RR 1.19**  (1.12 to 1.26) | 802 (8 studies) | ⊕⊕⊝⊝ **low**^2,3^ |  |
|  | **786 per 1000** | **935 per 1000** (880 to 990) |  |  |  |  |
|  | **Moderate** | |  |  |  |  |
|  | **755 per 1000** | **898 per 1000** (846 to 951) |  |  |  |  |
| **MA frequency - Q.d** | **Study population** | | **RR 1.18**  (1.1 to 1.26) | 578 (6 studies) | ⊕⊕⊝⊝ **low**^2,4^ |  |
|  | **792 per 1000** | **935 per 1000** (872 to 998) |  |  |  |  |
|  | **Moderate** | |  |  |  |  |
|  | **755 per 1000** | **891 per 1000** (831 to 951) |  |  |  |  |
| **MA frequency - Q.o.d** | **Study population** | | **RR 1.21**  (1.08 to 1.35) | 224 (2 studies) | ⊕⊕⊝⊝ **low**^1,2^ |  |
|  | **768 per 1000** | **929 per 1000** (829 to 1000) |  |  |  |  |
|  | **Moderate** | |  |  |  |  |
|  | **761 per 1000** | **921 per 1000** (822 to 1000) |  |  |  |  |
| **Period of treatment** | **Study population** | | **RR 1.22**  (1.13 to 1.31) | 550 (6 studies) | ⊕⊕⊝⊝ **low**^2,4^ |  |
|  | **760 per 1000** | **927 per 1000** (859 to 996) |  |  |  |  |
|  | **Moderate** | |  |  |  |  |
|  | **747 per 1000** | **911 per 1000** (844 to 979) |  |  |  |  |
| **Period of treatment - 2 weeks** | **Study population** | | **RR 1.17**  (1.06 to 1.29) | 248 (2 studies) | ⊕⊕⊝⊝ **low**^1,2^ |  |
|  | **806 per 1000** | **944 per 1000** (855 to 1000) |  |  |  |  |
|  | **Moderate** | |  |  |  |  |
|  | **805 per 1000** | **942 per 1000** (853 to 1000) |  |  |  |  |
| **Period of treatment - 4 weeks** | **Study population** | | **RR 1.25**  (1.11 to 1.41) | 252 (3 studies) | ⊕⊕⊝⊝ **low**^2,5^ |  |
|  | **730 per 1000** | **913 per 1000** (810 to 1000) |  |  |  |  |
|  | **Moderate** | |  |  |  |  |
|  | **714 per 1000** | **892 per 1000** (793 to 1000) |  |  |  |  |
| **Period of treatment - 8 weeks** | **Study population** | | **RR 1.35**  (1.01 to 1.81) | 50 (1 study) | ⊕⊕⊝⊝ **low**^1,2^ |  |
|  | **680 per 1000** | **918 per 1000** (687 to 1000) |  |  |  |  |
|  | **Moderate** | |  |  |  |  |
|  | **680 per 1000** | **918 per 1000** (687 to 1000) |  |  |  |  |
| *The basis for the **assumed risk** (e.g. the median control group risk across studies) is provided in footnotes. The **corresponding risk** (and its 95% confidence interval) is based on the assumed risk in the comparison group and the **relative effect** of the intervention (and its 95% CI). **CI:** Confidence interval; **RR:** Risk ratio; | | | | | | |
| GRADE Working Group grades of evidence **High quality:** Further research is very unlikely to change our confidence in the estimate of effect.  **Moderate quality:** Further research is likely to have an important impact on our confidence in the estimate of effect and may change the estimate. **Low quality:** Further research is very likely to have an important impact on our confidence in the estimate of effect and is likely to change the estimate. **Very low quality:** We are very uncertain about the estimate. | | | | | | |
| ^1^ Downgraded one level owing to high risk of bias in the blinding of participants of the included study. ^2^ Downgraded one level owing to publication bias caused by small sample size. ^3^ Downgraded one level owing to high risk of performance bias across the included studies, of eight trials, one trial was at high risk of bias owing to allocation concealment and lack of blinding of participants.  ^4^ Downgraded one level owing to high risk of performance bias across the included studies, of six trials, one trial was at high risk of bias owing to allocation concealment and lack of blinding of participants.  ^5^ Downgraded one level owing to high risk of performance bias across the included studies, of three trials, one trial was at high risk of bias owing to allocation concealment and lack of blinding of participants. | | | | | | |

**Table. 2**

**Summary of findings: The influence of acupuncture type on efficacy and the possible use of acupuncture in combination with or as a substitute for drug therapy in patients with ulcerative colitis.**

| **The influence of acupuncture type on efficacy and the possible use of acupuncture in combination with or as a substitute for drug therapy in patients with ulcerative colitis.** | | | | | | |
| --- | --- | --- | --- | --- | --- | --- |
| **Patient or population:** patients with UC **Settings:** outpatient/ inpatient **Intervention:** MA/MA plus medicine/EA plus medicine | | | | | | |
| **Outcomes** | **Illustrative comparative risks* (95% CI)** | | **Relative effect (95% CI)** | **No of Participants (studies)** | **Quality of the evidence (GRADE)** | **Comments** |
|  | Assumed risk | Corresponding risk |  |  |  |  |
|  | **Control** | **MA/EA** |  |  |  |  |
| **MA vs medicine** | **Study population** | | **RR 1.18**  (1.11 to 1.25) | 824 (8 studies) | ⊕⊕⊝⊝ **low**^1,2^ |  |
|  | **791 per 1000** | **934 per 1000** (878 to 989) |  |  |  |  |
|  | **Moderate** | |  |  |  |  |
|  | **788 per 1000** | **930 per 1000** (875 to 985) |  |  |  |  |
| **MA/EA plus medicine vs medicine** | **Study population** | | **RR 1.28**  (1.17 to 1.4) | 432 (5 studies) | ⊕⊕⊝⊝ **low**^2,3^ |  |
|  | **722 per 1000** | **924 per 1000** (845 to 1000) |  |  |  |  |
|  | **Moderate** | |  |  |  |  |
|  | **710 per 1000** | **909 per 1000** (831 to 994) |  |  |  |  |
| **MA plus medicine versus medicine** | **Study population** | | **RR 1.26**  (1.13 to 1.4) | 320 (3 studies) | ⊕⊕⊝⊝ **low**^2,4^ |  |
|  | **731 per 1000** | **921 per 1000** (826 to 1000) |  |  |  |  |
|  | **Moderate** | |  |  |  |  |
|  | **735 per 1000** | **926 per 1000** (831 to 1000) |  |  |  |  |
| **EA plus medicine versus medicine** | **Study population** | | **RR 1.36**  (1.13 to 1.63) | 112 (2 studies) | ⊕⊕⊝⊝ **low**^2,5^ |  |
|  | **696 per 1000** | **947 per 1000** (787 to 1000) |  |  |  |  |
|  | **Moderate** | |  |  |  |  |
|  | **695 per 1000** | **945 per 1000** (785 to 1000) |  |  |  |  |
| **MA vs mesalazine** | **Study population** | | **RR 1.2**  (1.09 to 1.32) | 282 (3 studies) | ⊕⊕⊝⊝ **low**^2,4^ |  |
|  | **780 per 1000** | **936 per 1000** (850 to 1000) |  |  |  |  |
|  | **Moderate** | |  |  |  |  |
|  | **781 per 1000** | **937 per 1000** (851 to 1000) |  |  |  |  |
| **MA plus mesalazine vs mesalazine** | **Study population** | | **RR 1.27**  (1.07 to 1.5) | 124 (2 studies) | ⊕⊕⊝⊝ **low**^2,5^ |  |
|  | **726 per 1000** | **922 per 1000** (777 to 1000) |  |  |  |  |
|  | **Moderate** | |  |  |  |  |
|  | **725 per 1000** | **921 per 1000** (776 to 1000) |  |  |  |  |
| **MA plus (metronidazole + SASP) vs metronidazole plus SASP** | **Study population** | | **RR 1.13**  (1.05 to 1.21) | 372 (3 studies) | ⊕⊕⊝⊝ **low**^2,5^ |  |
|  | **839 per 1000** | **948 per 1000** (881 to 1000) |  |  |  |  |
|  | **Moderate** | |  |  |  |  |
|  | **823 per 1000** | **930 per 1000** (864 to 996) |  |  |  |  |
| **EA plus SASP vs SASP** | **Study population** | | **RR 1.36**  (1.13 to 1.63) | 112 (2 studies) | ⊕⊕⊝⊝ **low**^2,5^ |  |
|  | **696 per 1000** | **947 per 1000** (787 to 1000) |  |  |  |  |
|  | **Moderate** | |  |  |  |  |
|  | **695 per 1000** | **945 per 1000** (785 to 1000) |  |  |  |  |
| *The basis for the **assumed risk** (e.g. the median control group risk across studies) is provided in footnotes. The **corresponding risk** (and its 95% confidence interval) is based on the assumed risk in the comparison group and the **relative effect** of the intervention (and its 95% CI). **CI:** Confidence interval; **RR:** Risk ratio; | | | | | | |
| GRADE Working Group grades of evidence **High quality:** Further research is very unlikely to change our confidence in the estimate of effect.  **Moderate quality:** Further research is likely to have an important impact on our confidence in the estimate of effect and may change the estimate. **Low quality:** Further research is very likely to have an important impact on our confidence in the estimate of effect and is likely to change the estimate. **Very low quality:** We are very uncertain about the estimate. | | | | | | |
| ^1^ Downgraded one level owing to high risk of performance bias across the included studies, of eight trials, one trial was at high risk of bias owing to allocation concealment and lack of blinding of participants. ^2^ Downgraded one level owing to publication bias caused by small sample size. ^3^ Downgraded one level owing to high risk of performance bias across the included studies, of five trials, one trial was at high risk of bias owing to allocation concealment and lack of blinding of participants.  ^4^ Downgraded one level owing to high risk of performance bias across the included studies, of three trials, one trial was at high risk of bias owing to allocation concealment and lack of blinding of participants.  ^5^ Downgraded one level owing to high risk of bias in the blinding of participants of the included study. | | | | | | |

**Table. 3**

**Summary of findings: Is acupuncture safer than drugs in treating ulcerative colitis?**

| **Is acupuncture safer than drugs in treating ulcerative colitis?** | | | | | | |
| --- | --- | --- | --- | --- | --- | --- |
| **Patient or population:** patients with UC **Settings:** outpatient/ inpatient **Intervention:** MA/MA plus medicine | | | | | | |
| **Outcomes** | **Illustrative comparative risks* (95% CI)** | | **Relative effect (95% CI)** | **No of Participants (studies)** | **Quality of the evidence (GRADE)** | **Comments** |
|  | Assumed risk | Corresponding risk |  |  |  |  |
|  | **Control** | **MA/EA** |  |  |  |  |
| **MA/EA vs medicine** | **Study population** | | **RR 0.32**  (0.18 to 0.59) | 592 (6 studies) | ⊕⊕⊕⊝ **moderate**^1,2^ |  |
|  | **135 per 1000** | **43 per 1000** (24 to 80) |  |  |  |  |
|  | **Moderate** | |  |  |  |  |
|  | **133 per 1000** | **43 per 1000** (24 to 78) |  |  |  |  |
| **MA/EA + medicine vs medicine** | **Study population** | | **RR 0.72**  (0.35 to 1.49) | 310 (5 studies) | ⊕⊕⊝⊝ **low**^1,2^ |  |
|  | **303 per 1000** | **218 per 1000** (106 to 452) |  |  |  |  |
|  | **Moderate** | |  |  |  |  |
|  | **233 per 1000** | **168 per 1000** (82 to 347) |  |  |  |  |
| *The basis for the **assumed risk** (e.g. the median control group risk across studies) is provided in footnotes. The **corresponding risk** (and its 95% confidence interval) is based on the assumed risk in the comparison group and the **relative effect** of the intervention (and its 95% CI).  **CI:** Confidence interval; **RR:** Risk ratio; | | | | | | |
| GRADE Working Group grades of evidence **High quality:** Further research is very unlikely to change our confidence in the estimate of effect.  **Moderate quality:** Further research is likely to have an important impact on our confidence in the estimate of effect and may change the estimate. **Low quality:** Further research is very likely to have an important impact on our confidence in the estimate of effect and is likely to change the estimate. **Very low quality:** We are very uncertain about the estimate. | | | | | | |
| ^1^ Downgraded one level owing to high risk of bias in the blinding of participants of the included study. ^2^ Downgraded one level owing to publication bias caused by small sample size. | | | | | | |

**Table. 4**

**Summary of findings: Can MA plus medicine reduce Baron scores in patients with UC?**

| **Can MA plus medicine reduce Baron scores in patients with UC?** | | | | | | |
| --- | --- | --- | --- | --- | --- | --- |
| **Patient or population:** patients with UC **Settings:** inpatient **Intervention:** MA plus medicine | | | | | | |
| **Outcomes** | **Illustrative comparative risks* (95% CI)** | | **Relative effect (95% CI)** | **No of Participants (studies)** | **Quality of the evidence (GRADE)** | **Comments** |
|  | Assumed risk | Corresponding risk |  |  |  |  |
|  | **Control** | **MA/EA** |  |  |  |  |
| **Baron scores** |  | The mean baron scores in the intervention groups was **1.31 standard deviations higher** (1.03 to 1.58 higher) |  | 256 (2 studies) | ⊕⊕⊝⊝ **low**^1,2^ | SMD 1.31 (1.03 to 1.58) |
| *The basis for the **assumed risk** (e.g. the median control group risk across studies) is provided in footnotes. The **corresponding risk** (and its 95% confidence interval) is based on the assumed risk in the comparison group and the **relative effect** of the intervention (and its 95% CI). **CI:** Confidence interval; | | | | | | |
| GRADE Working Group grades of evidence **High quality:** Further research is very unlikely to change our confidence in the estimate of effect.  **Moderate quality:** Further research is likely to have an important impact on our confidence in the estimate of effect and may change the estimate. **Low quality:** Further research is very likely to have an important impact on our confidence in the estimate of effect and is likely to change the estimate. **Very low quality:** We are very uncertain about the estimate. | | | | | | |
| ^1^ Downgraded one level owing to high risk of performance bias across the included studies, of two trials, one trial was at high risk of bias owing to allocation concealment and lack of blinding of participants. ^2^ Downgraded one level owing to publication bias caused by small sample size. | | | | | | |
